# Networks of CD8^+^ T Cell Response Activation in Melanoma and Vitiligo

**DOI:** 10.3389/fimmu.2022.866703

**Published:** 2022-04-01

**Authors:** Keitaro Fukuda

**Affiliations:** ^1^ Department of Dermatology, Keio University School of Medicine, Tokyo, Japan; ^2^ Laboratory for Skin Homeostasis, RIKEN Center for Integrative Medical Sciences, Yokohama, Japan

**Keywords:** melanoma, vitiligo, melanocyte/melanoma-shared antigen, CD8+ T cell, keratinocyte, dendritic cell, JAK signaling, CD122

## Abstract

Melanoma is an aggressive skin cancer derived from melanocyte, which shows high response rate to cancer immunotherapy, such as immune checkpoint inhibitors (ICIs). Vitiligo is an autoimmune skin disease resulting from the destruction of melanocytes by autoreactive CD8^+^ T cells. Vitiligo induced by cancer immunotherapy is a favorable prognostic factor in patients with melanoma, and growing evidence supports the fact that melanocyte/melanoma-shared antigen (MSA)-specific CD8^+^ T cells infiltrated in the tumor (melanoma) and skin (vitiligo) microenvironment play pivotal roles in the prognosis of both diseases. Thus, cellular communications that promote MSA-specific CD8^+^ T cells recruitment, proliferation, and effector functions are now seen as key targets to enhance the efficacy of current therapies for both diseases. Here, we discussed recent advancements in illustrating immune signaling pathways and immune cell types that regulate migration, proliferation, and function of MSA-specific CD8^+^ T cells in melanoma and vitiligo; and future immunotherapeutic approaches that may enhance clinical outcomes of both diseases.

## Introduction

Melanoma is a highly aggressive skin cancer that is particularly immunogenic, evident by its ability to undergo spontaneous and cancer immunotherapy-induced regression. The advent of immune checkpoint inhibitors (ICIs), such as the anti-PD-1 antibody (Ab) has improved the prognosis of patients with metastatic melanoma. However, 5-year overall survival rate for patients with stage IV melanoma is limited to 40% of patients (1). Thus, new treatments with the ability to enhance the efficacy and durability of current cancer immunotherapies are needed.

Vitiligo is caused by the destruction of melanocytes by CD8^+^ T cells and can be induced by administering ICIs (also called vitiligo-like depigmentation) to patients with melanoma. Vitiligo and antitumor responses in melanoma reportedly depend on CD8^+^ T cells that recognize identical antigens, so-called melanocyte/melanoma-shared antigens (MSA), including Melan-A, tyrosinase, and premelanosome (PMEL), and can reduce melanoma mass and prevent tumor recurrence (2). Indeed, vitiligo induced by immunotherapy is strongly associated with response to ICI therapy and patient survival (3). These results suggest that MSA-specific CD8^+^ T cell responses promote vitiligo progression and can suppress melanoma progression and vice versa.

Several studies have demonstrated that the vitiligo disease activity and prognosis of cancer immunotherapy for melanoma correlate with the number and effector function of MSA-specific CD8^+^ T cells in the skin and melanoma, respectively (4–7). Furthermore, infiltration and proliferation of MSA-specific CD8^+^ T cells are associated with the number of MSA-specific CD8^+^ T cells. These factors and the effector function of MSA-specific CD8^+^ T cells are regulated by cellular communications in the skin and melanoma microenvironment. Thus, understanding networks of CD8^+^ T cell response provides opportunities for a new treatment strategy for vitiligo and melanoma.

## MSA-Specific Cd8^+^ T Cell Recruitment in Melanoma and Vitiligo

ICIs have limited efficacy for patients who have insufficient tumor antigen-specific CD8^+^ T cell infiltration of their tumor, a characteristic known as “cold tumor” (8). In contrast, a melanoma infiltrated by a large number of MSA-specific CD8^+^ T cells, referred to as a “hot tumor,” responds well to ICIs. Thus, converting “cold tumor” to “hot tumor” by inducing MSA-specific CD8^+^ T cell infiltration would provide a valuable adjunctive therapy to improve ICIs efficacy.

Translational research using hot tumors revealed that the spontaneous MSA-specific CD8^+^ T cell infiltration into tumors is facilitated by the recognition of tumor-derived DNA by the cytosolic cGAS-STING signaling pathway in tumor-infiltrating conventional type 1 dendritic cells (cDC1 TIDCs) (9–11). This leads to type I interferon (IFN) production by cDC1 TIDCs and promotes their migration to the tumor-draining lymph node, thereby priming MSA-specific CD8^+^ T cells and promoting their migration to the tumor *via* CXCL10 (11). In this setting, STING agonists are now being injected intratumorally in clinical trials to increase the efficacy of anti-PD-1 Ab treatment in patients with advanced melanoma (12, 13). However, 48% of cold tumor-type melanoma have aberrant activation of WNT/β-catenin signaling and lack cDC1 TIDCs (14). Therefore STING agonists, which stimulate the function of cDC1 TIDC, may not be effective in cold tumor-type melanoma. Thus, a treatment strategy that activates the STING-type I IFN pathway and increases cDC1 TIDCs would be optimal for combined therapy with ICIs.

Studies of human melanoma and melanoma mouse model revealed that NK cells contribute to the recruitment of cDC1 DCs into tumors, through the production of FLT3L, CCL5, and XCL1 (15, 16). In addition, the melanoma mouse model demonstrated that mobilization of cDC1 alone is insufficient in generating anti-tumor T cell response in tumors and that addition of the TLR3 agonist, which activates type I IFN signaling in cDC1, is necessary to enhance response to anti-PD-1 Ab (17). A randomized clinical trial that conducted FLT3L pre-treatment enhancement of responses to the DC vaccine in combination with the TLR3 agonist for metastatic melanoma found that the addition of FLT3L increased peripheral cDC1, cDC2, and plasmacytoid DCs. Furthermore, the increase of humoral and T-cell responses and activation of DCs, NK cells, and T cells was observed (18). These results suggest that anti-PD-1 Ab in combination with FLT3L and TLR3 agonist might be an effective treatment for cold tumor-type melanoma (19).

Similar to the STING signaling pathway, which is absent in melanoma 2 (AIM2) is a cytosolic double-stranded DNA sensor, activated by cytosolic DNA. AIM2 signaling leads to the production of inflammatory cytokines IL-1β and IL-18, eliciting a form of cell death called pyroptosis. Additionally, we found that DC expression of AIM2 within human melanoma correlates with poor prognosis, and AIM2 exerts an immunosuppressive effect within the melanoma (20). Therefore, vaccination with AIM2-deficient DCs improves the efficacy of adoptive T-cell therapy and anti-PD-1 Ab for “cold tumors.” This effect depends on STING activation and IFN-β production, leading to CXCL10-mediated recruitment of T cells into the tumor. Additionally, regulatory T cell (Treg) tumor infiltration was reduced, which was due to the loss of AIM2-dependent production of IL-1β and IL-18. These results suggest that Aim2-deficient DC vaccination not only enhances immune responses to the tumor by activating STING, but also modulates IL-1β and IL-18 production, resulting in synergistic therapeutic responses. Thus, anti-PD-1 Ab in combination with Aim2-suppressed DC vaccine (intravenous or intra-tumoral injection) or Aim2 siRNA (intra-tumoral injection) will be a new treatment strategy for melanoma.

Gene expression analysis of both human vitiligo skin and vitiligo mouse model skin revealed the upregulation of IFN-γ-specific signature, including chemokines CXCL10 and CXCL9, and their receptor CXCR3, which is expressed on T cells (21). In the vitiligo mouse model, blockade of IFN-γ, IFNGR (receptor of IFN-γ), JAK1/2 (downstream intracellular signal transducer of IFNGR), CXCL10, and CXCR3 suppressed the MSA-specific CD8^+^ T cell skin infiltration, thereby not only preventing vitiligo progression but also reversing vitiligo, indicating that the IFN-γ-chemokine axis is a potential therapeutic target (22). Functional studies in the CXCL10 reporter vitiligo model mice and single-cell RNA sequence of the human vitiligo skin revealed that antigen-presenting cells, including DCs and macrophages, upregulated CXCL10 to the greatest extent on a per-cell basis, which was similar to melanoma (4, 11, 23). However, since keratinocytes are abundant in the skin, they produced the bulk of CXCL10, and were predominantly responsible for MSA-specific CD8^+^ T cell recruitment in vitiligo. These results indicated that targeting IFN-γ signaling in keratinocytes, which can be done by topical treatment, could be effective for treating vitiligo.

## The Role of IFN-γ Signaling and JAK Inhibitor in Melanoma and Vitiligo

Given that IFN-γ-JAK1/2-CXCL10 signaling in keratinocytes plays a major role in MSA-specific CD8^+^ T cell recruitment in vitiligo, the efficacy of a topical JAK1/2 inhibitor, ruxolitinib cream, was evaluated by a prospective randomized phase 2 trial (24). After 24 weeks of treatment, those who received ruxolitinib cream at 1.5% twice daily showed a significantly higher proportion of patients achieving a 50% or higher improvement from baseline in the facial Vitiligo Area Scoring Index (F-VASI50) than those receiving placebo (45% vs. 3%, p = 0.0001). These results suggest that topical JAK inhibitors might be new effective treatment for vitiligo, and phase 2 or 3 studies of several topical JAK inhibitors are ongoing based on these results (25).

In cancer, IFN-γ is known to promote tumor-antigen specific CD8^+^ T cell responses by upregulating the MHC class I on tumor cells, thereby increasing their antigen recognition (26). However, at the same time in the tumor microenvironment, where the persistence of antigen and inflammation occurs, IFN-γ can upregulate immune checkpoint ligands, such as PD-L1 on cancer cells that leads CD8^+^ T cells to enter a state called “T cell exhaustion.” Exhausted CD8^+^ T cells increase the expression of immune checkpoint receptors, such as PD-1, LAG3, and TIM3, as well as loss of cytokines, such as IFN-γ and TNF-α, production in a hierarchical manner and lose their effector function by time (27). Based on this notion, a study demonstrated that anti-PD-1 Ab lose their efficacy over time because of the expression of other immune checkpoint receptors like TIM-3 (28). This suggests that inhibiting multiple immune checkpoint receptors would be required for durable, long-term responses in ICIs.

Recently, the single-cell RNA-seq analysis of melanoma demonstrated that expression of the IFN-γ hallmark gene set (IFNG.GS) is predominantly expressed in T cells, NK cells, and macrophages, whereas IFN-stimulated genes (ISGs) resistance signature (ISG.RS) is predominantly expressed in melanoma cells (29, 30). Furthermore, functional assays using mice with melanoma that are resistant to anti-CTLA-4 Ab plus radiation therapy revealed that inhibition of tumor IFN-γ signaling decreases ISGs in cancer cells, while it increases the number and IFN-γ production of CD8^+^ T cells and activates NK cells in melanoma. Thus, ISGs expressed by cancer and immune cells oppose each other, and IFNG.GS (immune cell)/ISG.RS (cancer cell) ratio correlates with the response of ICIs (30, 31). Furthermore, delayed administration of the JAK inhibitor after starting the anti-CTLA-4 Ab improved its antitumor response by decreasing expression of multiple immune checkpoint ligands on melanoma cells. In contrast, co-administration of the JAK inhibitor and the anti-CTLA-4 Ab did not improve antitumor response compared to anti-CTLA-4 Ab monotherapy (31). Therefore, these results suggest that ICI lead-in before JAK inhibitor combination extends the response of ICI in melanoma.

## PD-1/PD-L1 in Melanoma and Vitiligo

Melanoma can be classified into four groups by PD-L1 expression on melanoma cells and the presence or absence of tumor-infiltrating lymphocytes (TILs). Among the four groups, 38% of melanoma express PD-L1 and exhibit TIL infiltration, and this type of melanoma is most likely to respond to the anti-PD-1 Ab since many CD8^+^ TILs in this melanoma express PD-1 and are in an exhausted T cell state (32). PD-1/PD-L1 ligation suppresses the proliferation and effector function of CD8^+^ TILs (33), and anti-PD-1 Ab could partially reinvigorate the antitumor immune response of MSA-specific CD8^+^ TILs by upregulating the production of IFN-γ and TNF-α, thereby increasing its proliferation rate (34, 35). However, approximately 25% of patients with melanoma treated with the anti-PD-1 Ab relapse after long-standing objective response by acquired resistance (36). Whole-exome sequencing of human melanoma tissues and the human melanoma cell line revealed that acquired resistance can result from loss-of-function mutations of IFNGR1, IFNGR2, JAK1, JAK2, STAT1, and IRF3 from the IFN-γ signaling pathway and β2M from antigen presentation pathway (37, 38). Since the IFN-γ signaling pathway in melanoma cells induces tumor growth arrest and death, mutations of IFNGR1, IFNGR2, JAK1, JAK2, STAT1, and IRF3 lead to insensitivity to anti-tumor IFN-γ activity (37, 38). In addition, JAK1 mutants are insensitive to type I IFNs (IFN-α and IFN-β), since JAK1 plays a role in downstream signaling of both type I and II IFN receptors (39). Furthermore, functional studies of human melanoma cell lines and tissues revealed that the IFN-γ-IFNGR1/2-JAK1/JAK2-STAT1/STAT2/STAT3-IRF1 axis promotes PD-L1 expression (40). Thus, mutations of the IFN-γ signaling pathway in melanoma cells lead to downregulation of PD-L1, thereby evolving into CD8^+^ T cell resistant-PD-L1 negative lesions. In contrast, β2M mutants are sensitive to IFN-α, IFN-β, and IFN-γ on cell growth inhibition and PD-L1 expression but lose MHC class I expression, thereby impairing antigen presentation (39). Moreover, it was demonstrated that melanoma with JAK1/2 mutants also block MHC class I upregulation by IFN-γ, similar to that of β2M mutants (38, 39). Thus, melanoma escapes antitumor immune response of anti PD-1 Ab by altering IFN signaling and/or the antigen presentation machinery.

Recent studies using the melanoma mouse model demonstrated that JAK1/2 knockout resistance could be overcome with anti-PD-1 Ab in combination with the TLR9 agonist, which activates TLR9-type IFN signaling, thereby increasing infiltration of T cells and NK cells in the tumor (39). β2M knockout resistance was able to overcome by anti-PD-1 Ab in combination with bempegaldesleukin (CD122 preferential IL-2 agonist) by activating cytotoxicity of NK cells and CD4^+^ TILs (39). Based on these results, clinical trials evaluating the TLR9 agonist or CD122 preferential IL-2 agonist in combination with anti-PD-1 Ab, for melanoma, are now ongoing (41, 42).

In contrast to melanoma, immunohistochemical analysis of human vitiligo tissues and *in vitro* experiments using human melanocytes showed that melanocytes derived from non-lesional vitiligo skin showed no PD-L1 upregulation upon IFN-γ exposure, while other skin cells displayed significant PD-L1 expression after exposure (43). These results suggest that melanocytes in vitiligo have low protection against MSA-specific CD8^+^ T cells and that manipulating PD-1/PD-L1 signaling might have therapeutic potential in vitiligo. In corroboration with the hypothesis, a report demonstrated that i.p. injection of PD-L1 fusion protein reversed hair depigmentation *via* increased Tregs in PMEL mouse, whose CD8^+^ T cells are all specific for PMEL and spontaneously developed hair depigmentation by perifollicular infiltration of PMEL-specific CD8^+^ T cells (44). Based on these findings, targeting the PD-1/PD-L1 axis can be effective as a treatment strategy for vitiligo.

## Survival Of MSA-Specific Cd8^+^ T Cell In Melanoma And Vitiligo

Although vitiligo is reversible by treatment, 40% of the patients experience relapse within the first year after treatment stops (45). Vitiligo typically recurs at the same location, indicating that MSA-specific memory CD8^+^ persists in the skin and permits disease reactivation after treatment terminates. Indeed, several groups reported that MSA-specific memory CD8^+^ T cells are highly enriched in the vitiligo lesion compared to blood, and the majority of these cells expressed CD69 and CD103, which are both markers of skin resident memory T cells (T_RM_) (46–48). Furthermore, T_RM_ cells in vitiligo was reported to express CD49a and CD49a^+^ CD8^+^ T_RM_ cells upregulate cytotoxic molecules, such as IFN-γ, perforin, and granzyme B when exposed to IL-15 (49). Reports demonstrated that IL-15 signaling mediates proliferation and survival of CD8^+^ T_RM_, and the number of CD8^+^ T_RM_ in the epidermis significantly reduced in IL-15-deficient mice compared to wild-type mice (50). Collectively, these results suggest that MSA-specific CD8^+^ T_RM_ is responsible for vitiligo homeostasis and IL-15 signaling is a potential therapeutic target.

The IL-15 receptor is composed of CD122 (which can be shared by the IL-2 receptor when paired with CD25), CD215, and CD132 (the common γ chain) and form a trimeric receptor (51). In vitiligo lesions, CD122 and CD132 are expressed on MSA-specific CD8^+^ T_RM_, whereas CD215 is expressed on keratinocytes with an ability to present IL-15 to T cells in trans (48). Based on this notion, a study using the vitiligo mouse model demonstrated that anti-CD122 Ab depletes CD8^+^ T_RM_ from vitiligo, thereby achieving durable repigmentation when administered either systemically or locally in the skin. Notably, both human and mouse MSA-specific CD8^+^ T_RM_ in vitiligo lesion showed higher expression of CD122 on a per cell basis and a higher proportion of CD122 expressing MSA-specific CD8^+^ T_RM_ compared to host CD8^+^ T_RM_, suggesting that targeting CD122 preferentially affects autoreactive T cells while leaving most endogenous T cell populations intact (48). Since systemic JAK inhibitors did not deplete MSA-specific CD8^+^ T_RM_ in the vitiligo mouse model (52), the anti-CD122 Ab could be a more durable and safer treatment strategy for vitiligo.

In contrast to T_RM_, recirculating central memory T cells (T_CM_) can migrate back and forth through circulation to tissues, such as the skin. T_CM_ expresses sphingosine-1-phosphate (S1P) receptor; thus, FTY720, a S1P receptor modulator prevents T_CM_ to egress from lymph nodes, thereby inhibiting T_CM_ recruitment to the skin. Interestingly, FTY720 treatment achieved repigmentation in a vitiligo mouse model. These results indicated that CD8^+^ T_RM_ cooperates with CD8^+^ T_CM_ to maintain vitiligo lesions, and the S1P1 modulator can be a new treatment strategy for vitiligo (53).

Like vitiligo, the role of CD8^+^ T_RM_ and CD8^+^ T_CM_ in melanoma has been investigated by many reports. Treating a melanoma-expressing OVA-bearing mouse by i.p. vaccination of recombinant vaccinia virus expressing OVA (rVACV-OVA) to generate CD8^+^ T_RM_ and CD8^+^ T_CM_, or only CD8^+^ T_RM_ by i.p. vaccination of rVACV-OVA with i.p. injection of FTY720, it was shown that both CD8^+^ T_RM_ and CD8^+^ T_CM_ infiltrated in melanoma are sufficient to mediate anti-tumor immunity, and they can synergize with each other (54). Another study revealed that approximately 40% of mice transplanted with melanoma in the epidermis remained free of macroscopic skin lesions for more than five months; however, intravital imaging revealed that these mice frequently harbored melanoma cells, and these cells were dynamically surveyed by CD8^+^ T_RM_. Depletion CD8^+^ T_RM_ triggered tumor outgrowth in a proportion (~20%) of mice with occult melanomas. These results suggest that CD8^+^ T_RM_ promotes a durable melanoma–immune equilibrium and suppresses melanoma progression (55).

Since CD8^+^ T_RM_ proliferation depends on IL-15 signaling, bempegaldesleukin (NKTR-214), an engineered IL-2 cytokine prodrug that provides sustained activation of the IL-2 pathway with a bias to the CD122, which works as a CD122 agonist, can promote proliferation of CD8+ T_RM_. Functional assay using the melanoma mouse model and the human melanoma tissues revealed that NKTR-214 expands, maintaining effector CD8^+^ T cells and depleting Tregs by effector CD8^+^ T cell-derived IFN-γ and TNF-α (39, 56). This CD122 agonist is now being injected intravenously in clinical trials to increase the efficacy of anti-PD-1 Ab treatment in patients with advanced melanoma and showing promising results (42).

## Conclusion

**Figure 1:**
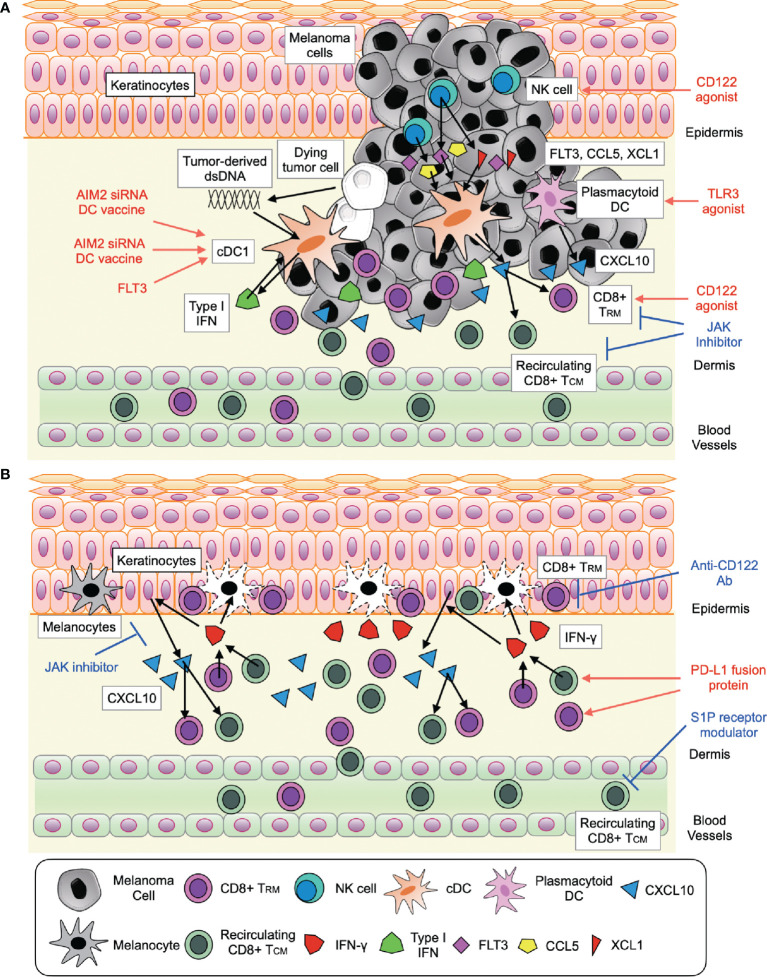
Signaling pathways involved in CD8^+^ T cell response activation and future therapeutic approaches in melanoma **(A)** and vitiligo **(B)**. **(A)** The interaction of (i) CD8^+^ T cell (CD8^+^ T_RM_ and recirculating CD8^+^ T_CM_) with cDC1 and plasmacytoid DC through type I IFN-CXCL10 signaling (ii) NK cell with cDC1 through FLT3, CCL5, and XCL1. **(B)** The interaction of CD8^+^ T cell (CD8^+^ T_RM_ and recirculating CD8^+^ T_CM_) with keratinocytes through IFN-γ-JAK signaling and IL-15 (CD122) signaling.

Melanoma is an aggressive skin cancer with a poor prognosis. Since, durable responses to cancer immunotherapies are limited to 40% of patients, improving clinical outcomes is imperative. Translational research to date has provided an insight into the networks that activate CD8^+^ T cell immune response in melanoma, providing a potential treatment strategy that enhance anti-tumor immunity of anti-PD-1 Ab, such as STING agonist, AIM2 siRNA, and combination therapy of FLT3L and TLR3 agonist that target TIDCs, JAK inhibitor for exhausted CD8^+^ T cells, and CD122 agonist for CD8+ TRM and NK cells in tumor (**Table 1**). Since melanoma and vitiligo are in a ying–yang relationship, opposite treatments to melanoma, such as anti-CD122 Ab and PD-L1 fusion protein are now focused as a new treatment strategy for vitiligo (**Table 1**). However, JAK inhibitor was both beneficial to both melanoma and vitiligo. Seemingly, these slight differences in cellular communications and responses in melanoma and vitiligo are partially attributable to the ratio of melanoma cells to immune cells is much higher than the ratio of melanocyte to immune cells in vitiligo (**Figures 1A, B**). However, it should still be noted understanding multiple aspects of CD8+ T cell immune response involved in melanoma and vitiligo would lead to better treatments and contribute toward a better prognosis in both diseases.

**Table 1:** Future Approaches for Targeting CD8+ T cell Immune Response in Melanoma and Vitiligo.

Disease	Target Function	Target Cell	Target	Examples Under Investigation	Reference
Melanoma	Promotion of CD8^+^ T cell Migration	Dendritic cells	STING-Type I Signaling	STING Agonist + Anti-PD-1 Ab	(12, 13)
		Dendritic cells	TLR3/STING -Type I Signaling	FLT3+ TLR3 agonistt + Anti-PD-1 Ab	(19)
		Dendritic cells	STING-Type I + AIM2-Signaling	AIM2 siRNA DCnVaccine + Anti-PD-1Ab	(20)
		Plasmacytoid Dendritic cells	TLR9-Type I Signaling	TLR9 Agonist + Anti-PD-1 Ab	(39, 41)
	Stimulation of CD8^+^ T cell Proliferation	CD8^+^ T_RM_ + NK cells	IL-15 Signaling	CD122 Agonist + Anti-PD-1 Ab	(39, 42, 56)
	Activation of CD8^+^ T cell Effector Function	CD8^+^ T cells	Immune Checkpoint	JAK Inhibitor (Oral) + ICI	(30, 31)
Vitiligo	Inhibtion of CD8^+^ T cell Migration	Keratinocytes	IFN-γ-JAK1/2-Signaling	JAK Inhibitor (Topical & Oral)	(24, 52)
		Recirulating T_CM_	S1P receptor	S1P recptor modulator	(53)
	Suppression of CD8^+^ T cell Proliferation	CD8^+^ T_RM_	IL-15 Signaling	Anti-CD122 Ab	(48)
	Suppression of CD8^+^ T cell Effector Function	CD8^+^ T cell	Immune Checkpoint	PD-L1 Fusion Protein	(44)

Ab, antibody; T_RM_, resident memory T cells; ICI, immune checkpoint inhibitor; TCM, central memory T cells; S1P, sphingosine-1-phosphate.

## Author Contributions

The author confirms being the sole contributor of this work and has approved it for publication.

## Funding

This work was supported by Grant-in-Aid for Scientific Research (C) Grants 21K08356 (to KF) from the Ministry of Education, Culture, Sports, Science and Technology, Japan.

## Conflict of Interest

KF has filed a patent that covers AIM2 siRNAs and their use to treat melanoma.

## Publisher’s Note

All claims expressed in this article are solely those of the authors and do not necessarily represent those of their affiliated organizations, or those of the publisher, the editors and the reviewers. Any product that may be evaluated in this article, or claim that may be made by its manufacturer, is not guaranteed or endorsed by the publisher.
